# Development and Validation of a Coping Strategies Scale for Use in Chinese Contexts

**DOI:** 10.3389/fpsyg.2022.845769

**Published:** 2022-03-23

**Authors:** Jian Zhao, Elaine Chapman, Stephen Houghton, David Lawrence

**Affiliations:** Graduate School of Education, University of Western Australia, Perth, WA, Australia

**Keywords:** coping strategies, Chinese university students, COVID-19, exploratory factor analysis, confirmatory factor analysis

## Abstract

Individuals’ coping strategies have a profound effect on how well they respond to negative life events. Despite this, most coping strategies instruments that are available currently have been developed exclusively in Western contexts. In the present study, a Coping Strategies Scale (CSS) for use with Chinese participants was developed and validated based on responses from 734 Chinese university students (334 male, 399 female, 1 other). Results supported a seven-factor structure for the CSS, which included the dimensions of Withdrawal, Positive Adaptation, Problem-solving, Disengagement, Prosocial Focus, Seeking Emotional Support, and Self-regulation. The results supported the validity of the seven-factor CSS in terms of its content, associated response processes, internal structure, and relationships with other variables. Based on these results, the CSS provides a psychometrically sound instrument for assessing the coping strategies used by Chinese adults in confronting potentially adverse psychological events.

## Introduction

The World Health Organization [WHO] (2022) declared COVID-19 as a “Public Health Emergency of International Concern” on the 30 January 2020, and as a global pandemic on 11 March 2020. By 7 March 2021, there had been 445,096,612 confirmed cases, including 5,998,301 deaths across the world. In addition to its effects on physical health, widespread concerns have been raised about the potential impact of the pandemic on the mental health of citizens across the world. Alarming evidence of adverse mental health outcomes including high levels of anxiety, depression, posttraumatic stress disorder, and psychological distress has been published in several countries (for reviews, see Octavius et al., 2020; Rajkumar, 2020; Xiong et al., 2020). China, the country in which the first confirmed case was reported, has been no exception to this trend, with various reports suggesting serious mental health consequences of the pandemic on Chinese residents (Yue et al., 2020; Wang et al., 2020; Zhang and Ma, 2020).

The mental health effects of the COVID-19 pandemic on individuals, however, may vary with the strategies that individuals use to deal with the crisis. The coping strategies that individuals adopt in confronting negative life events have been found to have a significant impact on how well they fare through those events (Skinner et al., 2003; Jopp and Schmitt, 2010). Not all coping strategies are equally effective (Alonso-Tapia et al., 2016), and these can either reduce or worsen the short-term emotional impact of negative events, as well as their impact on long-term mental, and subsequently, physical, health (Skinner et al., 2003). Coping strategies are, however, also considered to be malleable, in that individuals who have a tendency to approach adverse events in a maladaptive or ineffectual way can learn to adopt more effective strategies when they confront subsequent events.

Given the learnability of coping strategies, both mental health professionals and researchers could benefit from access to a culturally relevant measure of individuals’ coping strategies as a basis for assessment and intervention. While various instruments to measure coping strategies are already available (e.g., the Ways of Coping Checklist by Folkman and Lazarus, 1980; The Coping Orientation to Problems Experienced by Carver et al., 1989), these have been developed almost exclusively using samples from Western countries. Cultural values have been found to have a significant effect on the coping strategies used by individuals (Aldwin, 2004; Connor, 2016; Powell and Wegmann, 2018). It is, therefore, possible that the instruments developed in Western contexts will not capture important coping strategies used by individuals in other countries. In light of this gap, the present study aimed to develop and validate an instrument suitable for assessing coping strategies in Chinese adults.

### Existing Instruments to Measure Individuals’ Coping Strategies

Lazarus and Folkman (1984) defined coping as “constantly changing cognitive and behavioral efforts to manage specific external and/or internal demands that are appraised as taxing or exceeding the resources of the person” (p. 141). Classification systems for different coping strategies vary widely, with Skinner et al. (2003) identifying more than 100 different systems that had been used in the field. Amongst these, both Lazarus and Folkman’s scheme (which divides strategies into problem- and emotion-focused approaches), and Endler and Parker’s (1990, 1994) tripartite system (task-oriented, emotion-oriented, and avoidance strategies) have been adopted widely, but various other categorizations have also been published (e.g., Billings and Moos, 1981, 1984; Carver et al., 1989). This section reviews existing instruments that have been widely adopted in the field, only one of which was developed in the Chinese context.

The Ways of Coping Checklist (WCCL) was developed by Folkman and Lazarus (1980) to measure the strategies that people use in specific stressful encounters. The original WCCL comprises 68 items, organized into seven subscales that measure defensive coping; information-seeking; problem-solving; palliation; inhibition of action; direct action; and magical thinking. Participants are asked to think about the most stressful events they experienced in the previous month and then indicate which strategies they used in dealing with this event (e.g., “Made a plan of action and followed it”—yes/no). Although the WCCL has been used in various studies, its factor stability, clinical generalizability and construct validity have attracted critical comment from several authors (Vitaliano et al., 1985; Carver et al., 1989; Amirkhan, 1990; Parker et al., 1993). The WCCL is also relatively lengthy, thereby increasing risk of survey fatigue (O’Reilly-Shah, 2017), particularly if it forms part of a battery of instruments used in an empirical study.

The Ways of Coping Questionnaire (WCQ) is an adaptation of the original WCCL (Lazarus and Folkman, 1984; Folkman and Lazarus, 1988), but uses a four-point rating scale (“not used at all” to “used a great deal”) instead of a yes/no format. Minor revisions to specific items within the WCCL were also made in developing the WCQ, to improve question clarity. The WCQ comprises 66 items, which fall into eight factors: confrontive; distancing; self-controlling; seeking social support; accepting responsibility; escape-avoidance; planful problem-solving; and positive reappraisal (Folkman and Lazarus, 1985; Folkman et al., 1986). The WCQ has been used widely within different samples and across different types of stressors. However, validity evidence has been inconsistent across studies. Senol-Durak et al. (2011) investigated the psychometric properties of the WCQ with two samples of Turkish university students (*n*_1_ = 472; *n_2_* = 485) as well as a sample of community members (*n*_3_ = 416), and found support for a seven-factor structure with acceptable reliability coefficients. Other studies, however, have been less favorable. For example, Edwards and O’Neill (1998) used confirmatory factor analysis to compare alternative factor structures for the WCQ with a group of business school graduates (*n* = 654) in the United States, and found poor fits for the proposed structure (e.g., average Tucker-Lewis Indices and Comparative Fit Indices of 0.65 and 0.67, respectively), as well as low internal consistencies (most failing to reach 0.70). The WCQ is also approximately the same length as the original WCCL.

The Coping Orientation to Problems Experienced (COPE) measure was designed to assess different ways in which people respond to stress (Carver et al., 1989), and includes 60 items which are categorized into 13 subscales—active coping; planning; suppression of competing activities; restraint coping; seeking of instrumental social support; emotional social support seeking; positive reinterpretation; acceptance; denial; turning to religion; venting emotions; behavioral disengagement; and mental disengagement. Participants are asked to rate how frequently they use each type of strategy (e.g., “I’ve been turning to work or other activities to take my mind off things”) on a 1 (I haven’t been doing this at all) to 4 (I’ve been doing this a lot) scale. The COPE has been translated into different languages and its psychometric properties have been evaluated extensively in Western contexts. In general, favorable results have been generated (Sica et al., 1997). However, like the WCCL and the WCQ, the COPE is relatively long at 60 items.

Carver (1997) subsequently developed a 28-item version of the COPE (the Brief COPE), which was initially validated with a group of 168 community residents who had been affected by a hurricane. This initial study indicated that the Brief COPE exhibited similar properties to the original version. Validation studies into the brief COPE since, however, have produced mixed results with respect to the factor structure of the instrument across cultures. Both Muller and Spitz (2003) and García et al. (2018) confirmed the 14-factor structure of the Brief COPE after translating the items into French and Spanish, respectively. However, studies in other contexts have reported varying factor structures, including a two-factor structure based on responses from female nurses in the United Arab Emirates (Rahman et al., 2021); a three-factor structure based on responses from British adults (Ingledew et al., 1996); a four-factor structure based on college students in an American university (Cramer, 2019); a six-factor based on responses of people living with HIV in China (Su et al., 2015); and a seven-factor model based on responses from international university students in America (Miyazaki, 2008).

Endler and Parker (1990) developed the 48-item Coping Inventory for Stressful Situations (CISS) to include16 items in each of three subscales (task-oriented, emotion-oriented and avoidance-oriented coping strategies). Respondents rate how often they use each strategy listed in the instrument (e.g., “Talk to someone whose advice I value”) on a five-point scale ranging from 1 = “Not at all” to 5 = “Very much.” Endler and Parker (1994) evaluated the properties of the CISS and reported high internal consistencies ranging from 0.83 to 0.90 across the subscales. The CISS has also been validated in different countries such as Japan (e.g., Furukawa, 1997), and found to exhibit similar internal consistencies in these contexts. Validation studies on the CISS have, however, suggested that the factor structure may vary across cultural contexts (e.g., Choi et al., 2017).

Endler and Parker (1994, 1999) also developed a short form of the CISS (CISS-21), which has since been translated and evaluated in several languages, including Turkish (Boysan, 2012), Urdu (Rodriguez-Diaz et al., 2014), and Chinese (Li et al., 2017). While a number of these evaluations have yielded similar results to those reported for the English version, both the factor structures and the internal consistencies have varied somewhat across studies. For example, in Li et al.’s evaluation based on 972 Chinese university students, Cronbach’s alphas as low as 0.65 were reported for one of the subscales. Furthermore, despite the fact that the CISS-21 has been translated to Chinese, it was not originally developed with Chinese participants, and may as a result exclude strategies that are used by individuals within this context.

The Simplified Coping Style Questionnaire (SCSQ) is the only coping strategies measure the authors could locate that was developed based on Chinese participants. Xie (1998) developed the 20-item SCSQ based on Folkman and Lazarus’s (1980) WCQ. The SCSQ incorporates two dimensions: positive coping styles (12 items—e.g., “Try to find different methods to solve problems”) and negative coping styles (8 items—e.g., “Try to forget about it”). Respondents are required to rate how often they use each strategy based on a four-point scale (“never” to “very often”). Xie (1998) examined the properties of the SCSQ with a sample of 20 Chinese university students and found high test-retest and Cronbach’s α coefficients. Despite its obvious potential advantages in terms of brevity and practicality, no explicit rationale was proffered for the selection of items included within the SCSQ, or for the overall negative/positive classification of the items. For example, it is arguable as to whether the strategy “Try to forget about it” is actually negative in all contexts (e.g., this strategy could actually be positive in situations that are entirely out of the individual’s control). Furthermore, the instrument has never been validated on a large sample of participants.

### Aim of the Present Study

Given that no widely validated CSSs for use in Chinese contexts could be located by the authors, the current study aimed to develop such an instrument and validate it in a large group of Chinese university students. The instrument was designed to be used in a variety of adverse circumstances (i.e., it asks about the strategies that participants generally use when confronting adverse events). However, it was validated against the backdrop of the global COVID-19 pandemic, having formed part of a larger survey on Chinese university students’ mental health during the pandemic. Therefore, participants in the validation study are likely to have used this event as a recent reference point when they completed the questionnaire.

## Method

### Participants

The Coping Strategies Scale (CSS) was completed by participants as part of a larger (195 item) survey. Participants were Chinese students enrolled in various university institutions across mainland China. Over 1,000 such students entered the survey, but following the data screening to remove partially completed surveys and instances of clearly disengaged responses (e.g., participants who missed five or more items; put the same answer for all questions; or finished the entire survey in less than 5 min), only 734 responses were retained for analysis.

Using a computer-generated list of random numbers, we then divided this sample of 734 responses into two groups for the validation exercise. Group A comprised 367 students, including 168 males (45.80%) and 198 females (54.00%) and one (0.30%) who indicated “other” for gender. Group B also comprised 367 students, including 166 males (45.2%) and 201 females (54.8%). The ages of the respondents ranged from 16 to 48 years, with a mean of 22.20 years (*SD* = 4.16) from Group A and a mean of 22.08 years (*SD* = 3.82) from Group B. For Group A, 22.30% of students were married or in a relationship as compared with 26.20% of students from Group B.

### Instrument

The rationale for constructing a scale and operationalizing coping strategies was that different coping strategies used by people have been found related to their mental health (Ito and Matsushima, 2017; Saxon et al., 2017; Tsaras et al., 2018). The CSS scale, therefore, is intended to be used both as an outcome measure in what coping strategies people tend to use in a specific stressful context, and as a predictor variable on people’s mental health. The instrument was developed based on literature that was published in English journals. All items were written in both Chinese and English forms concurrently. The first author is a speaker of both Chinese and English, and thus was able to write the item statements in both forms. The CSS comprised 30 specific strategies/items, with respondents asked to rate how frequently they use each of the strategies listed using a five-point frequency rating scale (Never = 1, Rarely = 2, Sometimes = 3, Often = 4, Always = 5). These were designed to assess 10 types of coping strategies: Opportunities for Growth (Growth); Identifying Alternatives (Alt); Problem Solving (Prob); Prosocial Focus (Prosoc); Seeking Professional Help (Prof); Seeking Emotional Support (Emot); Self-Regulation (Sreg); Disengagement (Diseng); Denial (Denial); and Withdrawal (WithD). A list of all CSS instrument statements can be found in Table 1. Means and standard deviations for all items, based on the two subgroups of *n* = 367 as described above, are also presented in Table 1.

**TABLE 1:** Item statements and descriptive statistics (Groups A and B, *n* = 734) for the coping strategies scale (CSS) and validation instruments.

Label	Statement	Group A	Group B
Growth1	I see the event as a chance to reflect on life (e.g., re-appraise what is important). 我会把它当成是一个反思生活的机会（例如，重新思考什么对我来说才是重要的）。	3.23 (0.92)	3.18 (0.92)
Growth2	I see the event as a chance for development or growth. 我会把它当成是一个自我发展或成长的机会。	3.27 (0.90)	3.21 (0.87)
Growth3	I focus on the good things that could come out of the event. 我会专注于这件事可能带来的好处。	3.17 (0.95)	3.20 (0.95)
Alt1	I take the opportunity to learn new skills. 我会借此机会学习新技能。	3.35 (0.91)	3.36 (0.86)
Alt2	I take the opportunity to develop new interests/hobbies. 我会借此机会发展新的兴趣/爱好。	3.14 (0.92)	3.12 (0.95)
Alt3	I see it as an opportunity to do things in a different way. 我会把它看作一个以不同方式做事的机会。	3.30 (0.84)	3.22 (0.92)
Prob1	I search for information (e.g., on the internet) on how to solve any problems that the situation brings. 我会搜索（比如上网）有关如何解决这个事件或情况的信息。	3.58 (0.98)	3.60 (0.99)
Prob2	I analyze the situation carefully to come up with the best way to deal with it. 我会认真分析情况，以提出最佳处理方法。	3.57 (0.90)	3.47 (0.89)
Prob3	I make a plan about how to solve any problems the event brings in a step-by-step way. 我会制定计划以便逐步解决这件事所带来的问题。	3.30 (0.95)	3.30 (0.94)
Prosoc1	I think of ways to help others who are worse off than myself. 我会想办法帮助那些境况比我差的人。	3.05 (0.94)	3.09 (0.91)
Prosoc2	I become more involved in community activities designed to help those in need. 我会更多地参与能够帮助需要帮助的人的社区活动。	2.91 (0.98)	2.92 (0.99)
Prosoc3	I think about ways to get involved with charity work. 我会想办法参与慈善工作。	2.78 (1.03)	2.86 (1.00)
Prof1	I consult people who know more about the problem on the best ways to address it. 想那些对这个问题有着更多了解的人咨询以便找到最佳的解决办法。	3.27 (0.94)	3.24 (0.88)
Prof2	I ask people who had similar experiences for their advice. 向有类似经验的人寻求建议。	3.39 (0.97)	3.36 (0.89)
Prof3	I seek help from professionals about what to do next. 向专业人员寻求帮助。	3.13 (0.99)	3.17 (0.94)
Emot1	I share my feelings with family members for emotional support. 我会与家人分享我的情绪以获得情感支持。	3.11 (1.06)	3.16 (1.00)
Emot2	I share my feelings with friends who will understand. 我会和能够理解我的朋友分享我的情绪。	3.46 (0.94)	3.50 (0.88)
Emot3	I share my feelings with others who are going through similar experiences. 我会和跟我具有相似经历的人分享我的情绪。	3.16 (1.02)	3.25 (0.90)
Sreg1	I tell myself regularly not to give up. 我会定期告诉自己不要放弃。	3.33 (0.93)	3.40 (0.94)
Sreg2	I find ways to cheer myself up when I feel down. 当我情绪低落时，我想办法振作起来。	3.57 (0.91)	3.60 (0.91)
Sreg3	I engage in relaxation exercises to ensure I remain calm. 我会进行一些轻松的运动使自己保持镇定。	3.22 (1.00)	3.31 (1.00)
Diseng1	I try not to look at or hear any information relevant to the event or situation. 我尽量不看或不听和这个事件或情况有关的任何信息。	2.98 (0.97)	3.12 (0.98)
Diseng2	I try to think about anything but the event. 我会想一些其他的事情。	3.28 (0.93)	3.35 (0.88)
Diseng3	I fill up my days with other activities to avoid thinking about the issue. 我会给自己安排很多活动以避免有时间想起这件事。	3.05 (0.99)	3.12 (0.95)
Denial1	I refuse to believe that the problem exists. 我拒绝相信这件事的存在。	2.27 (1.14)	2.36 (1.15)
Denial2	I try to pretend the situation is not real. 我假装这件事不是真的。	2.21 (1.14)	2.27 (1.14)
Denial3	I carry on as normal, and don’t respond to what has happened. 我像往常一样生活，对这件事不作回应。	2.57 (1.04)	2.58 (1.12)
WithD1	I stay away from people. 我会远离人群。	2.61 (1.17)	2.53 (1.13)
WithD2	I take every chance I can to be alone. 我会抓住一切机会独处。	2.82 (1.05)	2.84 (1.03)
WithD3	I turn off my phone so that no one can contact me. 我会关掉电话让任何人都联系不到我。	2.21 (1.13)	2.18 (1.12)

To provide further evidence related to concurrent validity two further instruments were completed by all participants. The first was a measure of well-being levels. Different coping strategies employed by individuals have been found to relate to well-being levels. Specifically, problem-solving coping strategies, positive reappraisal, support-seeking have been found consistently to relate to higher levels of well-being, while avoidance coping strategies have been found to be associated with lower well-being levels (Gustems-Carnicer and Calderón, 2013; Sagone and De Caroli, 2014; Freire et al., 2016). The World Health Organization—Five Well-Being Index (WHO-5) (World Health Organization [WHO], 1998) was used as the well-being measure in the study. This measure includes five items, each of which requires respondents to rate their wellbeing levels on a six-point Likert scale ranging from 0 (at no time) to 5 (all of the time). A large body of research has affirmed the sound psychometric properties of the WHO-5 as a measure of subjective well-being (Krieger et al., 2013; Downs et al., 2017; White and Van Der Boor, 2020).

The second measure used to assess the concurrent validity of the CSS was a measure of respondents’ self-efficacy for solving challenging problems they encountered in their lives. Self-efficacy is defined as “people’s beliefs about their capabilities to produce designated levels of performance that exercise influence over events that affect their lives” (Bandura, 1994, para. 4). As noted by Bandura (2006), instruments to measure self-efficacy must be constructed to reflect the specific tasks or activities of interest. The brief measure used in the current study was constructed based on Bandura’s (2006) item construction recommendations, using a bi-polar statement format:

• When problems arise in my life, I often worry that I won’t be able to solve them vs. I always believe I can solve any problem that confronts me in life.
• I worry a lot about my ability to cope with life’s challenges vs. I’m always very confident about my ability to do well in difficult life situations.
• I am often fearful about my ability to solve problems vs. I believe I can solve most of the problems I confront if I set my mind to it.
• Compared with other people, I think I tend to struggle more with unexpected problems vs. Compared with most people, I think I cope pretty well with unexpected problems.

Respondents were provided with a 7-point scale for the self-efficacy measure (-3 to +3). As indicated, all items in this scale related specifically to respondents’ confidence in their ability to cope with and address problems within their lives. Previous studies have indicated that high levels of such types of self-efficacy are associated with active coping strategies such as problem-solving (Taiwo, 2015; Manuel Morales-Rodriguez and Manuel Perez-Marmol, 2019), while low levels of such self-efficacy are associated with passive coping strategies such as avoidance (Taiwo, 2015).

### Data Collection

Convenience sampling and snowball sampling were used for data collection. After obtaining ethics approval from the authors’ University Ethics Committee, information about the survey was distributed via WeChat. The CSS was completed online through the Qualtrics platform. An anonymous link to the survey was sent to a large group of Chinese university students via WeChat. Those who completed the survey were also encouraged to forward the link to their circles of friends on WeChat to attract more participants. All of the participants and potential participants were informed that it would take them approximately 5–16 min to finish the survey, but that they could stop and resume filling in the survey if necessary. This was done to ensure that participants filled in the survey carefully, rather than in a rush to reach the end. No special incentives or rewards were offered for participation. All participants completed the survey voluntarily and were able to withdraw from the survey at any point prior to submission.

### Data Analysis

Exploratory factor analysis (EFA) and confirmatory factor analysis (CFA) were used to evaluate the internal structure of the CSS. EFA is a statistical technique used to conduct a preliminary analysis of the constructs which are needed to account for patterns of correlation among measures when there is no expectation regarding the factorial structure of an instrument (Fabrigar and Kan, 2018). CFA is a statistical technique used to verify the hypothesized relationships between observed indicators and their underlying latent variables (Randall and Jung, 2018). In this study, EFA was conducted by IBM SPSS V27 and CFAs were conducted by LISREL V8.80.

## Results

The validation study was guided by Messick’s (1989) seminal treatise on validity evidence. In this treatise, validity was defined as “an integrated evaluative judgment of the degree to which empirical evidence and theoretical rationales support the adequacy and appropriateness of inferences and actions based on test scores or other modes of assessment” (p. 13). This definition is aligned also with the revised definition of validity adopted since 1999 by the American Educational Research Association (AERA), American Psychological Association (APA) and National Council on Measurement in Education (NCME), in their *Standards for Educational and Psychological Testing* (American Educational Research Association [AERA] et al., 2014).

The instrument validation for the CSS addressed four of the five types of evidence stipulated by Messick (1989, 1995) in his re-conceptualization of validity. These were: evidence related to the content of the instrument; evidence of the response processes needed to complete the instrument; evidence of the instrument’s internal structure; and evidence of relations between the instrument’s scores and those on conceptually related variables. The only form of evidence stipulated by Messick (1989) that was not collected in the present study related to the consequences of the instrument’s use. This form of evidence requires extensive use of the instrument over a longer period of time, and fell beyond the scope of the present study.

### Validity Evidence Based on Test Content

To develop items for the CSS, a comprehensive search was first conducted to identify all coping strategies questionnaires published previously for use with adults either in Western or Chinese contexts. All elements of these existing instruments were considered for inclusion to ensure that the CSS was as comprehensive as possible. This included all instruments reviewed previously in the current paper (i.e., the WCC and revised WCC; the COPE and Brief COPE; the CISS; and the SCSQ), and those that appeared only in specific papers, such as the instruments published by Stone and Neale (1984), Amirkhan (1990), Jiang (1999), Garnefski et al. (2001), Heppner et al. (2006), Maxwell and Siu (2008), and Aitken (2011). After the CSS was developed by the first two authors, it was sent to two professors in the area of mental health, who commented upon the wording and content of the items. Revisions to the wordings were then made based on this feedback, and the changes were translated to the Chinese version.

### Validity Based on Response Processes

After the content of the instrument was deemed to be suitable in light of the expert reviews obtained, cognitive process interviews were then conducted with four Chinese university students on a one-on-one basis, and all the interviews were conducted in Chinese. In these sessions, participants were asked to read each item in the questionnaire carefully and indicate to the interviewer (the first author) any items or questions that confused them. This process was undertaken to ensure that the response processes associated with completing the questionnaire were aligned with the intent of the instrument, and to identify any items that generated confusion or ambiguity. Based on the feedback obtained, some further adjustments were then made to the wording of specific items.

### Validity Evidence Based on Internal Structure

To evaluate the internal structure of the CSS, an exploratory factor analysis (EFA) was conducted on data from Group A (*n* = 367), and a confirmatory factor analysis (CFA) performed on the data from Group B (*n* = 367). Cronbach’s alphas were also computed for each group to evaluate the internal consistency of the CSS. IBM SPSS V26 and LISREL V8.80 were used to conduct the EFAs and CFAs, respectively. A principal components analysis (PCA) extraction method was used in the EFA conducted on the Group A data, given that this was intended to be a preliminary analysis to indicate the number of dimensions that were tenable for the CSS. As the components were expected to be relatively independent of one another, these were rotated to approximate simple structure using the Varimax approach.

Preliminary screening analyses indicated no significant violations of EFA assumptions associated with linearity, normality, factorability, and case-to-item ratio. Rotated loadings are presented in Table 2. The scree plot suggested that a seven-component solution would provide the best fit to the data, though the eigenvalue of the seventh component fell marginally below 1. A comparison of the six- and seven-component solutions obtained indicated that the seven-component solution, which accounted for 68.50% of the total item variance, was more conceptually tenable, and better aligned with the theoretical structure. The six-component solution was similar to the seven-component, except that the two of the three self-regulation items (Sreg 1 and Sreg3) loaded with the Prosocial Focus items, while the remaining self-regulation item (Sreg 2) loaded with the Disengagement items. It was thus decided that the seven-component solution would be retained for interpretation, though the superiority of this model over the six-component solution obtained was also tested directly through the CFAs conducted on the Group B data.

**TABLE 2:** Communalities and rotated factor loadings for the coping strategies scale (CSS) (Group A data, *n* = 367).

Item label	*h* ^2^	Rotated component loadings						
		1. Positive adaptation	2. Withdrawal	3. Problem-solving	4. Prosocial focus	5. Seeking Emotional support	6. Disengagement	7. Self-regulation
Alt1	0.75	**0.79**	–0.05	0.19	0.22	–0.06	0.06	0.15
Growth3	0.62	**0.75**	0.07	0.15	0.01	0.19	0.03	–0.02
Growth2	0.70	**0.75**	0.01	0.19	0.03	0.16	0.13	0.25
Alt3	0.59	**0.68**	0.07	0.27	0.18	0.06	0.03	0.10
Growth1	0.56	**0.66**	0.03	0.17	–0.08	0.17	0.16	0.18
Alt2	0.63	**0.66**	0.02	0.08	0.41	0.03	0.13	0.07
WithD1	0.78	–0.05	**0.84**	0.14	–0.12	–0.13	0.09	0.08
WithD3	0.71	0.01	**0.78**	–0.08	0.29	–0.07	0.08	0.03
WithD2	0.80	0.10	**0.76**	0.21	–0.14	–0.34	0.09	0.18
Denial1	0.80	0.04	**0.75**	–0.13	0.34	0.27	0.14	–0.10
Denial2	0.78	0.02	**0.74**	–0.10	0.36	0.24	0.17	–0.07
Denial3	0.59	0.06	**0.68**	0.04	0.09	0.24	0.22	–0.09
Prob1	0.61	0.18	–0.05	**0.74**	0.04	–0.04	0.12	0.10
Prof2	0.72	0.20	0.04	**0.74**	0.21	0.28	0.15	0.14
Prof1	0.69	0.18	0.10	**0.70**	0.25	0.27	0.06	0.12
Prof3	0.65	0.21	0.07	**0.63**	0.29	0.29	0.16	–0.09
Prob2	0.62	0.42	–0.08	**0.60**	0.02	0.05	0.00	0.27
Prob3	0.61	0.35	0.04	**0.52**	0.25	0.14	–0.10	0.36
Prosoc2	0.78	0.19	0.18	0.27	**0.77**	0.14	0.09	0.16
Prosoc3	0.73	0.11	0.24	0.22	**0.75**	0.15	0.05	0.16
Prosoc1	0.62	0.18	0.19	0.34	**0.58**	0.19	–0.01	0.26
Emot1	0.73	0.13	0.05	0.13	0.11	**0.77**	–0.08	0.30
Emot2	0.65	0.22	–0.02	0.30	0.10	**0.66**	0.20	0.17
Emot3	0.66	0.18	0.07	0.30	0.26	**0.63**	0.24	0.07
Diseng2	0.73	0.16	0.14	0.20	–0.02	0.10	**0.75**	0.25
Diseng1	0.69	–0.01	0.33	0.01	0.00	0.05	**0.74**	0.17
Diseng3	0.66	0.28	0.24	0.10	0.22	0.09	**0.68**	–0.03
Sreg2	0.75	0.30	–0.04	0.18	0.09	0.20	0.19	**0.73**
Sreg1	0.71	0.20	0.03	0.18	0.25	0.30	0.16	**0.67**
Sreg3	0.63	0.23	0.03	0.16	0.37	0.06	0.33	**0.55**

*The bolded elements represent the loadings of the items on the factors they were deemed to define.*

Based on the seven-factor solution, the *Prosocial Focus*, *Seeking Emotional Support*, *Disengagement*, and *Self-regulation* items all fell into their own separate components as predicted (Components 4, 5, 6 and 7, respectively). The other three empirically obtained EFA components incorporated two of the original item clusters. These were Component 1 (which incorporated Opportunities for Growth and Identifying Alternatives items); Component 2 (which incorporated the original Withdrawal and Denial items); and Component 3 (which incorporated the original Seek Professional Help and Problem-Solving items). These three larger components were labeled *Positive Adaptation*, *Withdrawal*, and *Problem-solving*, respectively. The soundness of the seven-component structure was further affirmed by the moderate to high internal consistencies obtained for each of the components using the Group A data, with αs of 0.88, 0.86, 0.86, 0.75, 0.86, 0.77 and 0.79 for the Withdrawal, Positive Adaptation, Problem-solving, Disengagement, Prosocial Focus, Seeking Emotional Support, and Self-regulation subscales, respectively.

The data screening analyses for the Group B data again confirmed that all relevant assumptions for factor analysis had been met. Four CFA models were conducted on these data to cross-validate the results obtained in the EFA based on the Group A data. In the first model, all 30 CSS items were loaded on one factor. The second model was based on the two-factor model (Problem-Focused vs. Emotion-Focused strategies) suggested by Lazarus and Folkman (1984). In the two-factor model, the original Problem-Solving and Seek Professional Help items loaded on one factor (representing the Problem-Focused dimension), and all others loaded on another (representing the Emotion-Focused dimension). The third and fourth models were based on the six- and seven-factor alternative structures suggested by the EFAs on Group A. The change in χ^2^ among the models was used to evaluate whether the model fit statistics for the four models were significantly different. Fit indices for the four models tested are shown in Table 3.

**TABLE 3:** Fit indices for three models of the coping strategies scale (CSS) (Group B data, *n* = 367).

Model	χ^2^	*df*	χ^2^/*df*	NNFI	CFI	SRMR	RSMEA
One-factor	4931.30	405	12.18	0.77	0.79	0.14	0.17
Two-factor	4717.67	404	11.67	0.78	0.81	0.14	0.17
Six-factor	1656.74	390	4.25	0.91	0.92	0.10	0.09
Seven-factor	1278.87	384	3.33	0.93	0.94	0.07	0.08

Based on the Δχ^2^ statistics, the seven-factor solution aligned with the EFA results was clearly the best-fitting model. Specifically, there was a significant increase in χ^2^ from the seven-factor to the one-factor model, Δχ^2^(21) = 3652.43, *p* < 0.001; from the seven-factor to the two-factor model, Δχ^2^(20) = 3438.80, *p* < 0.001; and from the seven-factor to the six-factor model, Δχ^2^(6) = 377.87, *p* < 0.001. The sound fit of the seven-factor model was further affirmed by the other fit indices obtained (Kline, 2005, 2016; Hooper et al., 2008). These included the ratio of the chi-square statistic to the model degrees of freedom (χ^2^/*df*), which measures the discrepancy between the sample and fitted covariance matrices, taking sample size into account (values ≤ 5 indicating acceptable model fit); the Non-Normed Fit Index (NNFI), which measures the relative fit of the proposed model to the null model (values ≥ 0.90 indicating acceptable model fit); the Comparative Fit Index (CFI), which measures the relative fit of the proposed model to the null model, but is less sensitive to sample size than the NNFI (values ≥ 0.90 indicating acceptable model fit); the Standardized Root Mean Square Residual (SRMR), which measures the difference between the residuals of the sample covariance matrix and the hypothesized model (values ≤ 0.08 indicating acceptable model fit); and the Root Mean Square Error of Approximation (RSMEA), which measures the discrepancy between the hypothesized model and the population covariance matrix (values ≤ 0.08 indicating reasonable model fit).

**FIGURE 1:**
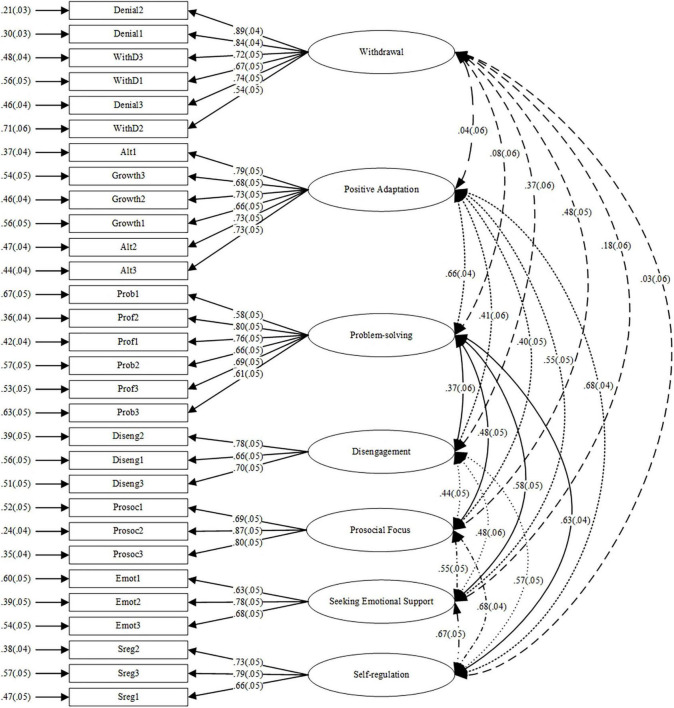
Path diagram for the seven-factor model of the coping strategies scale (CSS).

Moderate to high internal consistencies were also obtained for each component of the instrument, with αs of 0.88, 0.87, 0.84, 0.75, 0.83, 0.73 and 0.76 for the Withdrawal, Positive Adaptation, Problem-solving, Disengagement, Prosocial Focus, Seeking Emotional Support, and Self-regulation subscales, respectively. Based on the Cronbach’s αs, four subscales exhibited high internal consistency (>0.80), while the other three were acceptable (>0.70) within the Group B data. Coefficients for the paths between each of the items and their respective latent factors are shown in Figure 1.

### Correlations With External Variables

To provide further validity evidence on the instrument, information on two variables that had been found previously to correlate with coping strategies (age and education level) were also collected. Deasy et al. (2014) found that students aged 27 years or older used escape avoidance strategies (which align broadly with the Withdrawal strategies in the CSS) less frequently, and positive reappraisal (which align broadly with the Positive Adaptation strategies in the CSS) more frequently than did younger students. With respect to education levels, Sandover et al. (2015) found that undergraduate medical students favored “distancing” and “escape avoidance” (which aligns with the Withdrawal subscale of the CSS) as coping strategies in times of stress, while graduate medical students favored “planful problem solving” (which aligns with the Problem-Solving subscale of the CSS). Ickes et al. (2015) also found that undergraduate students were more likely than postgraduate students to use different forms of escapism (which again align broadly with the Withdrawal strategies in the CSS) as coping strategies.

In the current study, multivariate analyses of variance (MANOVAs) were performed to determine whether there were any significant differences in the use of coping strategies across different age groups and education levels. Means for the subgroups are shown in Table 4. All evaluations of conformity to MANOVA assumptions produced satisfactory results.

**TABLE 4:** Descriptive statistics and ANOVA outcomes for age and education level.

Variable	Subgroup	Positive adaptation	Withdrawal	Problem-solving	Prosocial focus	Seeking emotional support	Disengagement	Self-regulation
Age	Under 21^A^	3.16 (0.73)*^C^	2.54 (0.87)^†C^	3.29 (0.71)*^C^	3.01 (0.83)^†B^	3.24 (0.76)	3.20 (0.75)	3.33 (0.78)*^C^
	22–26^B^	3.22 (0.69)	2.41 (0.90)	3.38 (0.74)	2.84 (0.88)^†A^	3.24 (0.84)	3.09 (0.82)	3.40 (0.85)
	27 or over^C^	3.42 (0.71)*^A^	2.28 (0.88)^†A^	3.57 (0.68)*^A^	2.81 (0.84)	3.45 (0.82)	3.01 (0.81)	3.63 (0.73)*^A^
Education level	Undergraduate	3.16 (0.73)*	2.53 (0.88)*	3.29 (0.71)*	2.98 (0.83)	3.23 (0.78)	3.17 (0.77)	3.32 (0.80)*
	Postgraduate	3.33 (0.68)*	2.33 (0.88)*	3.50 (0.71)*	2.84 (0.87)	3.36 (0.82)	3.08 (0.81)	3.55 (0.78)*

*^A/B/C^Differed significantly from at least one other mean at adjusted level, superscripts indicate means that differed.

*^†^Difference from at least one other mean approached significance at the adjusted level.*

Results from the MANOVAs indicated a significant difference on the linear composite CSS variable across the age groups, λ = 0.91, *F*(14, 1502) = 4.93, *p* < 0.001, partial η^2^ = 0.04, and the education levels, λ = 0.93, *F*(7, 746) = 8.44, *p* < 0.001, partial η^2^ = 0.07. Follow-up analyses of variance (ANOVAs) were thus performed to explore each of these significant multivariate effects in more detail. The Holm-Bonferroni correction procedure (see Holm, 1979) was applied in evaluating the ANOVA outcomes, to maintain the α level at 0.05 for each MANOVA.

The ANOVAs testing the effect of age group on coping strategies indicated significant differences across groups on four of the seven coping subscales: *Problem-Solving*, *Self-Regulation*, *Positive Adaptation*, and *Prosocial Focus*, *F*s(2, 757) ≥ 3.11, *p*s ≤ 0.01, partial η^2^ ≥ 0.01. The effect on *Withdrawal* also approached significance at the adjusted α level, *F*(2, 757) = 3.08, *p* = 0.02, partial η^2^ = 0.01, while the effects of age group on *Seeking Emotional Support* and *Disengagement* were not significant. Results of the Tukey *post-hoc* tests for age groups, conducted at the 0.01 level, showed that students under 21 had significantly lower scores than those who were 27 years or older for *Positive Adaptation* (*p* = 0.004), *Problem-Solving* (*p* = 0.002), and *Self-Regulation* (*p* = 0.004). The difference between students under 21 and those between 22 and 26 years approached significance for *Prosocial Focus* (*p* = 0.03), as did the difference between students under 21 and those 27 years or older for *Withdrawal* (*p* = 0.03). For the latter subscales, students under 21 had higher scores than did older students. The results obtained for Emotional Support and Withdrawal align with the findings of Deasy et al. (2014), and thus support the validity of the new CSS.

For education level, the ANOVAs again indicated significant differences across groups on four of the seven of the coping subscales: *Problem-Solving*, *Self-Regulation*, *Positive Adaptation*, and *Withdrawal*, *F*s(1, 752) ≥ 7.76, *p*s ≤ 0.01, partial η^2^ ≥ 0.01. The effects on *Prosocial Focus*, *Seeking Emotional Support*, and *Disengagement* were not significant. The significant differences identified indicated that undergraduate students reported using problem-solving, positive adaptation, and self-regulation less frequently, but relied more frequently on withdrawal as a strategy, than did postgraduate students. The latter results (specifically, those obtained for the *Problem-Solving*, *Positive Adaptation*, and *Withdrawal* subscales) align well with the results reported by Ickes et al. (2015) and Sandover et al. (2015), which again leads support to the construct validity of the new CSS.

To provide further evidence related to concurrent validity, relationships between the CSS subscales and those from the WHO-5 and self-efficacy instrument described previously were explored. These analyses indicated significant positive relationships between Positive Adaptation, Prosocial, Seeking Emotional Help, Self-regulation, Problem-Solving and Disengagement with both WHO-5 scores, all *r*s(725) > 0.15, *p*s < 0.001, and with self-efficacy scores, *r*s(725) > 0.29, *p*s < 0.001. The relationship between Disengagement scores and the WHO-5 was also positive, though the strength of the relationship was more modest, *r*(725) = 0.08, *p* = 0.03. Similarly, scores on the self-efficacy measure were positively related to CSS Disengagement scores, though more modestly than for other CSS subscale scores, *r*(725) = 0.18, *p* < 0.001. These results are consistent with the previous research cited previously. In contrast, scores on the Withdrawal subscale were not significantly correlated either with WHO-5 scores, *r*(725) = 0.07, *p* > 0.05, or with self-efficacy scores, *r*(725) = 0.03, *p* > 0.05. This pattern of results is also aligned with findings reported previously in the literature.

## Discussion

The results of this study provide strong support for the validity of the new 30-item CSS, in terms of its content, response processes, internal structure, and relations with other variables. Based on the results of the EFAs and CFAs, the CSS includes seven distinct types of coping strategies: Withdrawal (six items), Positive Adaptation (six items), Problem-solving (six items), Disengagement (three items), Prosocial Focus (three items), Seeking Emotional Support (three items), and Self-regulation (three items). Cronbach’s αs showed that four subscales had good internal consistency (>0.80), while three subscales had acceptably high reliability (>0.70) in both of the samples used. Age and education level also correlated with the CSS subscales in theoretically reasonable ways, providing further support for the validity of the CSS.

Previous research has found that coping strategies that individuals used while dealing with negative life events have a significant impact on how well they fare through those events (Skinner et al., 2003; Jopp and Schmitt, 2010). Not all coping strategies are equally effective (Alonso-Tapia et al., 2016), and some coping strategies used by individuals have been found play an important role in their mental health (Burns et al., 2016). The newly developed CSS allow people, especially those in Chinese context, have a better understanding of the coping strategies they tend to adopt while dealing with negative life events. This may also allow them to tailor their coping strategies gradually to better deal with negative events.

This study is not without limitations. First, the sample used in this study are Chinese university students and may not fully represent the general Chinese population. It would be useful to validate the instrument with Chinese adults outside the university sector. Second, convenience sampling and snowball sampling were used in the current study for cost-effective purpose, which may compromise the unbiased nature of the data. Further research can try to use random sampling method. Third, this single evaluation of the CSS is also not sufficient to provide evidence on its psychometric properties across diverse cultures. Thus, further research will be needed to ensure that the sound properties demonstrated in the current study hold in different participant groups. Should its properties be confirmed, future studies could also make use of the CSS to measure the coping strategies that are used by individuals across different kinds of negative life events. For example, it is entirely possible that the optimal coping strategies for individuals to adopt may vary across these events, depending on whether they are relatively controllable or fall entirely outside of the individual’s control. Having a means by which to assess such strategies could contribute significantly to research and clinical practices which focus on ensuring that individuals maintain a high level of positive mental health, even in the face of adversity.

## Data Availability Statement

The raw data supporting the conclusions of this article will be made available by the authors, without undue reservation.

## Ethics Statement

The studies involving human participants were reviewed and approved by the University of Western Australian Human Ethics Committee. The participants provided their online informed consent to participate in this study.

## Author Contributions

JZ: conceptualization, data curation, formal analysis, investigation, methodology, project administration, validation, visualization, writing—original draft, and writin—review and editing. EC: conceptualization, data curation, formal analysis, investigation, methodology, project administration, supervision, validation, visualization, writing—original draft, and writing—review and editing. SH and DL: conceptualization, methodology, supervision, and writing—review and editing. All authors contributed to the article and approved the submitted version.

## Conflict of Interest

The authors declare that the research was conducted in the absence of any commercial or financial relationships that could be construed as a potential conflict of interest.

## Publisher’s Note

All claims expressed in this article are solely those of the authors and do not necessarily represent those of their affiliated organizations, or those of the publisher, the editors and the reviewers. Any product that may be evaluated in this article, or claim that may be made by its manufacturer, is not guaranteed or endorsed by the publisher.
